# Recovering the truth about the first doctoral program in nursing in the North region

**DOI:** 10.1590/0034-7167.20257802302

**Published:** 2025-07-11

**Authors:** Gilsirene Scantelbury de Almeida, Zilmar Augusto de Souza, Nair Chase da Silva, Noeli das Neves Toledo, Maria Jacirema Ferreira Gonçalves

**Affiliations:** I Universidade Federal do Amazonas, Escola de Enfermagem de Manaus. Manaus, Amazonas, Brazil

We would like to begin this editorial by apologizing to our readers. An editorial is expected to provide innovative content that adds knowledge and prepares us for reading the articles that follow. However, this text has a different purpose: to correct information missing from the editorial published on February 3, 2025 entitled “An overview of the implementation of the first doctorate program in nursing in the Northern region” ^(1)^.

Although the aforementioned editorial deals with a subject of interest for the current historical moment, as defined by the Brazilian Journal of Nursing (In Portuguese*, Revista Brasileira de Enfermagem* - REBEn) in its guidelines for authors, regarding the types of articles considered, some clarification is necessary to avoid perpetuating information that some would consider to be a half-truth. Is there such a thing as a half-truth?

The editorial to which we refer deals with the implementation of the first academic doctoral course in nursing in the North region of *Universidade do Estado do Pará* (UEPA). In fact, this course belongs to PPGENF UEPA/UFAM, Graduate Program in Nursing in Broad Association between UEPA and *Universidade Federal do Amazonas* (UFAM). In other words, it is a program developed jointly by both higher education institutions^(2)^.

PPGENF UEPA/UFAM is not a recent initiative. Its first master’s class began 14 years ago, with one class in Manaus and another in Belém. Currently, the program has classes in Coari and Manaus (AM), and in Santarém and Belém (PA). The submission of the doctoral program to the Presentation of Proposals for New Courses of the Coordination for the Improvement of Higher Education Personnel was carried out in January 2023^(1)^ jointly by both institutions, resulting in the approval of the course, which began in the second quarter of 2024, with 16 doctoral students distributed equally between Amazonas and Pará.

The installation ceremony was held jointly, reaffirming the institutional partnership. Currently, the program has permanent faculty, collaborators and visitors, and is already preparing to launch a new call for proposals for 2025. It is about this doctoral course that the aforementioned editorial refrained from highlighting the history of the partnership and its essential contribution to the existence of the first doctoral course in the North region.

How can we explain that the information contained in the editorial refers only to an institution such as the one that holds the doctoral course, when the association is in the public domain and especially to those who work in it? How can we apologize for a “slip” if one of the documents used as a reference^(3)^ in the editorial refers to the course as a doctoral course associated between the two higher education institutions?

In fact, it is difficult to understand how an entire history of *stricto sensu* construction over 14 years can be subtracted, making invisible the participation of those who were side by side in the work so that the North region could emerge from anonymity in relation to research and the presence of graduate studies at master’s and doctoral levels.

The publication caused several discomforts: for the UFAM *Escola de Enfermagem de Manaus*, which had its name removed from such an important historical moment for the nursing course; for the professors, administrative technicians in education and graduate students, who also worked to deliver a doctoral course in nursing for the North region and found themselves excluded; for REBEn, one of our most respected publication vehicles for scientific articles, which, in good faith, published an editorial whose information does not correspond to the truth; for the scientific community, whose expectation of an editorial is, as we said initially, to prepare us for subsequent readings, and not to reestablish the truth, as we are doing at this moment.

Furthermore, the publication in its current form violates the ethical principles and institutional relations formally established for the existence of the course.

For these reasons, this editorial aims to reestablish the truth. We reiterate our commitment to transparency and academic ethics, and we hope you enjoy reading the articles presented below.

## References

[1] Frazão JM, Santana ME. (2025). An overview of the implementation of the first doctorate program in nursing in the Northern region. Rev Bras Enferm.

[2] Universidade do Estado do Pará (UEPA) (2023). Universidade Federal do Amazonas (UFAM). Apresentação de Propostas de Cursos Novos para o curso de doutorado acadêmico em enfermagem associado UEPA/UFAM.

[3] Conselho Federal de Enfermagem (Cofen) (2024). Primeiro doutorado em Enfermagem da Região Norte é implementado no Pará.

