# Supplementary material for: Recovering the truth about the first doctoral program in nursing in the North region
Source: Rev Bras Enferm. 2025 Jul 11;78(3):e780302. doi: 10.1590/0034-7167.20257802302 (PMC12252915; doi:10.1590/0034-7167.20257802302)
Supplement: Translation

# Translation: Restabelecendo a verdade sobre o primeiro doutorado em enfermagem da região Norte

**DOI:** 10.1590/0034-7167.20257802302pt

**Authors:** Gilsirene Scantelbury de Almeida, Zilmar Augusto de Souza, Nair Chase da Silva, Noeli das Neves Toledo, Maria Jacirema Ferreira Gonçalves

**Affiliations:** I Universidade Federal do Amazonas, Escola de Enfermagem de Manaus. Manaus, Amazonas, Brasil

Iniciamos este editorial pedindo desculpas aos leitores. De um editorial, espera-se um conteúdo inovador, que agregue conhecimento e nos prepare para a leitura dos artigos que se seguem. No entanto, este texto tem um propósito diferente: corrigir informações ausentes no editorial publicado em 3 de fevereiro de 2025 intitulado “O olhar sobre a implantação do primeiro doutorado em enfermagem da Região Norte” ^(1)^.

Embora o referido editorial trate sobre assunto de interesse para o momento histórico, como definido pela Revista Brasileira de Enfermagem (REBEn) nas orientações aos autores, no quesito tipos de artigos considerados, é necessário algum esclarecimento sob pena de se perpetuar uma informação que alguns considerariam como uma meia verdade. Será que existe meia verdade?

O editorial a que nos referimos trata da implantação do primeiro curso de doutorado acadêmico em enfermagem na região Norte da Universidade do Estado do Pará (UEPA). Na verdade, esse curso pertence ao PPGENF UEPA/UFAM, Programa de Pós-Graduação em Enfermagem em Associação Ampla entre a UEPA e a Universidade Federal do Amazonas (UFAM). Ou seja, trata-se de um programa desenvolvido conjuntamente por ambas as instituições de ensino superior^(2)^.

O PPGENF UEPA/UFAM não é uma iniciativa recente. Sua primeira turma de mestrado teve início há 14 anos, com uma turma em Manaus e outra em Belém. Atualmente, o programa mantém turmas em Coari e Manaus (AM), e em Santarém e Belém (PA). A submissão do doutorado à Apresentação de Propostas de Cursos Novos da Coordenação de Aperfeiçoamento de Pessoal de Nível Superior foi realizada em janeiro de 2023^(1)^, de forma conjunta, pelas duas instituições, resultando na aprovação do curso, que teve início no segundo trimestre de 2024, com 16 doutorandos distribuídos igualmente entre Amazonas e Pará.

A cerimônia de instalação foi realizada de maneira conjunta, reafirmando a parceria institucional. Atualmente, o programa conta com docentes permanentes, colaboradores e visitantes, e já se prepara para lançar um novo edital para 2025. É sobre esse curso de doutorado que o citado editorial se absteve de destacar o histórico da parceria e sua contribuição essencial para a existência do primeiro curso de doutorado na região Norte.

Como explicar que as informações contidas no editorial se refiram apenas a uma instituição como aquela detentora do curso de doutorado, quando a associação é de domínio público e principalmente dos que nele atuam? Como se desculpar por um “deslize” se um dos documentos utilizado como referência^(3)^ no editorial se reporte ao curso como doutorado associado entre as duas instituições de ensino superior?

De fato, é difícil de entender como toda uma história de construção *stricto sensu* durante 14 anos possa ser subtraída, invisibilizando a participação de quem esteve lado a lado na labuta, para que a região Norte saísse do anonimato em relação à pesquisa e à presença da pós-graduação em nível de mestrado e doutorado.

A publicação gerou vários desconfortos: para a Escola de Enfermagem de Manaus da UFAM, que teve seu nome subtraído de tão importante momento histórico para o curso de enfermagem; para os docentes, técnicos administrativos em educação e pós-graduandos, que também trabalharam para entregar um curso de doutorado em enfermagem para a região Norte e se viram excluídos; para a REBEn, um de nossos mais respeitados veículos de publicação de artigos científicos que, de boa-fé, publicou um editorial cujas informações não condizem com a verdade; para a comunidade científica, cuja expectativa de um editorial é, como dissemos inicialmente, preparar-nos para as leituras subsequentes, e não para restabelecer a verdade, como fazemos nesse momento.

Ademais, a publicação na forma em que se encontra fere os princípios éticos e as relações institucionais formalmente estabelecidas para a existência do curso.

Por essas razões, este editorial tem como objetivo restabelecer a verdade. Reiteramos nosso compromisso com a transparência e a ética acadêmica, e desejamos uma boa leitura dos artigos apresentados a seguir.

# Translation: Restableciendo la verdad sobre el primer programa de doctorado en enfermería en la región Norte

**DOI:** 10.1590/0034-7167.20257802302es

**Authors:** Gilsirene Scantelbury de Almeida, Zilmar Augusto de Souza, Nair Chase da Silva, Noeli das Neves Toledo, Maria Jacirema Ferreira Gonçalves

**Affiliations:** I Universidade Federal do Amazonas, Escola de Enfermagem de Manaus. Manaus, Amazonas, Brasil

Comenzamos este editorial disculpándonos con los lectores. De una editorial esperamos contenido innovador, que aporte conocimientos y nos prepare para leer los artículos siguientes. Sin embargo, este texto tiene un propósito diferente: corregir información faltante en el editorial publicado el 3 de febrero de 2025 titulado “Un vistazo a la implementación del primer doctorado en enfermería en la región Norte” ^(1)^.

Si bien el citado editorial aborda un tema de interés para el momento histórico, tal como lo define la Revista Brasileña de Enfermería (En portugués, *Revista Brasileira de Enfermagem* - REBEn) en las directrices para autores, en cuanto a los tipos de artículos considerados, son necesarias algunas aclaraciones, so pena de perpetuar informaciones que algunos considerarían una verdad a medias. ¿Existe una verdad a medias?

El editorial al que nos referimos trata sobre la implementación del primer curso académico de doctorado en enfermería en la región Norte de *Universidade do Estado do Pará* (UEPA). De hecho, este curso pertenece al PPGENF UEPA/UFAM, Programa de Postgrado en Enfermería en Amplia Asociación entre UEPA y *Universidade Federal do Amazonas* (UFAM). Es decir, es un programa desarrollado en conjunto por ambas instituciones de educación superior^(2)^.

El PPGENF UEPA/UFAM no es una iniciativa reciente. Su primera maestría comenzó hace 14 años, con una clase en Manaus y otra en Belém. Actualmente, el programa mantiene clases en Coari y Manaus (AM), y en Santarém y Belém (PA). La presentación del doctorado a la Presentación de Nuevas Propuestas de Curso de la Coordinación de Perfeccionamiento del Personal de Educación Superior fue realizada en enero de 2023^(1)^, de manera conjunta, por las dos instituciones, resultando en la aprobación del curso, que comenzó en el segundo trimestre de 2024, con 16 estudiantes de doctorado distribuidos equitativamente entre Amazonas y Pará.

El acto de instalación se realizó de manera conjunta, reafirmando la alianza institucional. Actualmente, el programa cuenta con profesores permanentes, colaboradores y visitantes, y ya se prepara para lanzar una nueva convocatoria para 2025. Es sobre este curso de doctorado que el mencionado editorial se abstuvo de resaltar la historia de la alianza y su contribución esencial para la existencia del primer curso de doctorado en la región Norte.

¿Cómo explicar que la información contenida en el editorial se refiera sólo a una institución como la que imparte el doctorado, cuando la asociación es de dominio público y principalmente para quienes allí trabajan? ¿Cómo disculparse por un “desliz” si uno de los documentos utilizados como referencia^(3)^ en el editorial se refiere al curso como un doctorado asociado entre las dos instituciones de educación superior?

De hecho, es difícil comprender cómo se pudo sustraer toda una historia de construcción *stricto sensu* a lo largo de 14 años, invisibilizando la participación de quienes estuvieron lado a lado en el trabajo, para que la región Norte pudiera salir del anonimato en relación a la investigación y a la presencia de cursos de posgrado a nivel de maestría y doctorado.

La publicación generó varios malestares: para la *Escola de Enfermagem de Manaus* de UFAM, que perdió su nombre en un momento histórico tan importante para la carrera de enfermería; para docentes, técnicos de educación administrativa y estudiantes de posgrado, que también trabajaron para impartir un curso de doctorado en enfermería en la región Norte y se vieron excluidos; para REBEn, uno de nuestros vehículos más respetados para la publicación de artículos científicos, que, de buena fe, publicó un editorial cuya información no coincide con la verdad; para la comunidad científica, cuya expectativa de un editorial es, como decíamos inicialmente, prepararnos para lecturas posteriores, y no restablecer la verdad, como lo hacemos en este momento.

Además, la publicación en su forma actual viola los principios éticos y las relaciones institucionales formalmente establecidos para la existencia del curso.

Por estas razones, este editorial pretende restablecer la verdad. Reiteramos nuestro compromiso con la transparencia y la ética académica, y esperamos que disfrute leyendo los artículos que se presentan a continuación.
